# Ideal cardiovascular health and the subclinical impairments of cardiovascular diseases: a cross-sectional study in central south China

**DOI:** 10.1186/s12872-017-0697-9

**Published:** 2017-10-18

**Authors:** Ya-Qin Wang, Chang-Fa Wang, Ling Zhu, Hong Yuan, Liu-Xin Wu, Zhi-Heng Chen

**Affiliations:** 1 Department of Health Management Centre, the Third Xiangya Hospital, Central South University, Tongzipo Road 138, Changsha, Hunan Province 410013 China; 2 Department of General Surgery, the Third Xiangya Hospital, Central South University, Changsha, Hunan Province China; 3 Department of Clinical Pharmacology Centre, the Third Xiangya Hospital, Central South University, Changsha, Hunan Province China; 4 Zhongguancun Xinzhiyuan Health Management Institute, Beijing, China

**Keywords:** Ideal cardiovascular health, Cardiovascular diseases, Subclinical atherosclerosis

## Abstract

**Background:**

Ideal cardiovascular health (CVH) is related to a low cardiovascular disease risk profile. This study aimed to investigate CVH metrics with both the biomarkers and markers of subclinical impairments of cardiovascular diseases (CVDs) in subjects from central south China.

**Methods:**

The ideal CVH score (non-smoking status; ideal body mass index; regular physical activity; healthy diet; and an optimal serum cholesterol, blood pressure, and glucose profile; 1 point for each; total score: 0–7) was collected from 3009 participants without a history of CVDs. Subclinical biomarkers were assessed using C-reactive protein, homocysteine, and microalbuminuria. The presence of subclinical disease markers was defined as having at least one of the following: increased carotid intima-media thickness, carotid plaque, left ventricular hypertrophy, left ventricular systolic dysfunction, or a reduced ankle-brachial index. The association of biomarkers and markers with the CVH score was evaluated using multivariate logistic regression and linear regression analyses.

**Results:**

Only 0.2% of the study participants met all 7 ideal CVH metrics (CVH score = 7). Compared to the female participants, the male participants had poorer CVH profiles and a higher incidence of subclinical lesions (*P* < 0.05). In the fully adjusted models, per 1-unit increase in the CVH score was inversely associated with the biomarker levels (β = -0.092 – -0.224, *P* < 0.05 for all) and the odds of the presence of markers (odds ratio, 0.808; 95% confidence interval, 0.755–0.865). Similar relationships were observed in the gender subgroups and were stronger in the females.

**Conclusion:**

A clear inverse association was observed between the biomarkers or markers of subclinical impairments and the CVH score in a central south Chinese population, implying the importance of ideal CVH for the primordial prevention of CVDs.

**Electronic supplementary material:**

The online version of this article (10.1186/s12872-017-0697-9) contains supplementary material, which is available to authorized users.

## Background

With urbanization, rapid economic development, and an ageing population, cardiovascular diseases (CVDs) have become the leading causes of morbidity and mortality in China, and the burden of these diseases is increasing at an alarming rate [1]. Consequently, a public health policy of primordial prevention has been advocated. CVDs are long-term chronic diseases that develop over decades from risk factors to subclinical impairment and then progress to clinical events [2]. Subclinical impairment, which is the presymptomatic stage of CVDs, is a fundamental precursor that can help identify early subjects who are at high risk for future CVDs and who can benefit the most from early interventions. This approach is also part of primary prevention. Some non-invasive established biomarkers and markers can detect and measure the subclinical stages of CVDs, such as C-reactive protein (CRP), carotid intima-media thickness (CIMT), carotid plaque, left ventricular (LV) hypertrophy, and LV systolic dysfunction.

In 2010, the American Heart Association (AHA) defined a simplified 7-item tool to promote ideal cardiovascular health (CVH), including both modifiable behaviours and objective measures, such as the following CVH metrics: physical activity, diet, body mass index (BMI), smoking, blood pressure, serum glucose and total serum cholesterol [3]. This approach emphasizes primordial prevention in which efforts are focused on preventing the development of risk factors, which is in contrast to primary or secondary prevention in which the focus is on the prevention of the first occurrence or recurrence of CVDs. Achieving a greater number of ideal CVH metrics has been reported to be associated with a lower risk of CVD incidence and mortality [4–7].

Few studies have investigated the joint effects of the 7 CVH metrics on the subclinical impairments of CVDs [8–10]. Most of these studies have investigated a single subclinical marker or a few makers (mainly aortic arch plaque, coronary artery calcification or common CIMT), and the studies have been relatively limited to US and European populations [8, 11]. Accordingly, the aim of the present study was to simultaneously investigate both the biomarkers and markers of subclinical impairments of CVDs with ideal CVH metrics in a community-based cohort in a Chinese population.

## Methods

### Study population

**Fig. 1:**
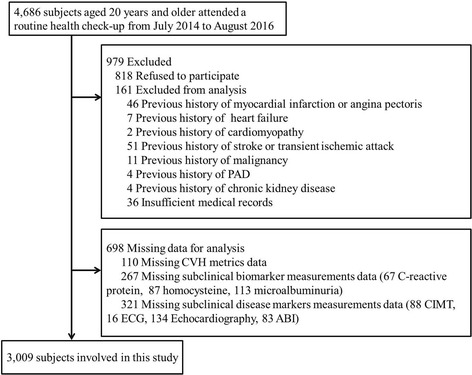
Study flowchart. PAD, peripheral artery disease; CVH, cardiovascular health; CIMT, carotid intima-media thickness; ECG, electrocardiogram; ABI, Ankle-brachial index

A cross-sectional study was performed. We retrospectively analysed clinical data from 4686 participants (aged 20 years and older) who attended a routine health check-up in the Health Management Centre of the Third Xiangya Hospital from July 2014 to August 2016. The population comprised white-collar workers (including teachers, professional technical personnel, civil servants, and administrative staff) living in Changsha, which is the capital city of Hunan province in central south China. The inclusion criteria were as follows: (1) no self-reported history of CVDs, including myocardial infarction or angina pectoris, heart failure, stroke or transient ischaemic attack; (2) no cardiomyopathy; (3) no malignancy; (4) no peripheral artery disease; (5) no chronic kidney disease; and (6) no data missing from the analysis. The study flowchart is presented in Fig. 1. Finally, 3009 subjects (2002 males, age range 24–85 years) were enrolled. The study protocol was approved by the Ethics Review Committee of our hospital. All participants agreed to study participation and provided informed consent.

### Assessment of CVH metrics

The CVH score was calculated according to the AHA guidelines by summing the 7 CVH metrics as dichotomous variables, with 0 reflecting a poor and 1 an ideal CVH status [3]. We used alternative measures for the dietary and physical activity metrics in this study. Ideal health behaviours and factors were defined as follows: no history of smoking or quit smoking >12 months previously, moderate physical activity for >150 min per week or vigorous physical activity for >75 min per week, 4 to 5 items of dietary status [with cereals and legumes as the basic food; ≥500 g (equivalent to 1 “Jin”) of vegetables and fruits/day, <100 g (equivalent to 2 “Liang”) of red meat/day, regular (in most weeks) intake of unprocessed fish and/or soybean products, and a preference for non-salty food] [12], BMI <25 kg/m^2^, untreated systolic BP <120 mmHg and diastolic BP <80 mmHg, untreated fasting blood glucose level < 5.6 mmol/L (100 mg/dL), and untreated total cholesterol level < 5.2 mmol/L (200 mg/dL). Participants who had 0–2, 3–4 and 5–7 health metrics at the ideal level were defined as having poor, intermediate and ideal CVH statuses, respectively [3].

Information concerning smoking, physical activity, and dietary intake was collected by questionnaires administered by well-trained staff blinded to the clinical data. The resting systolic blood pressure (SBP), diastolic blood pressure (DBP), and BMI were measured using standard methods [13]. The biochemical variables, including total cholesterol (TC) and fasting plasma glucose (FPG), were measured using an autoanalyser (Olympus, AU400, Japan) in the central laboratory of our hospital.

### Subclinical biomarker measurements

The three biomarkers represented the following distinct biological pathways: vascular inflammation state (CRP), vascular endothelial function and oxidative stress (homocysteine), and microvascular structural alteration (microalbuminuria). Blood samples were drawn by venepuncture after at least 10 h of overnight fasting. CRP was measured using a high-sensitivity nephelometry assay (Cias Latex CRP-H; Kanto Chemical, Tokyo, Japan). Homocysteine was measured using a chemiluminescent immunoassay (Centaur XP; Siemens, Deerfield, IL, USA). Urine was collected in the morning. Microalbuminuria was measured using kit reagents and the ProSpec nephelometric analyser (Dade Behring GMBH, Marburg, Germany).

### Subclinical disease marker measurements

Five subclinical atherosclerosis phenotypes were measured for this study. B-mode ultrasonography examinations of the carotid arteries were performed by an experienced sonographer using the Siemens Acuson Sequoia™ 512 Ultrasound System (Mountain View, CA, USA) with a 12-MHz (9–14) linear matrix array transducer with the patient in the supine position. CIMT was measured bilaterally on 3 segments: the distal common carotid artery (1 cm proximal to dilation of the carotid bulb), the carotid artery bifurcation, and the proximal internal carotid arteries (1-cm section of the internal carotid arteries immediately distal to the flow divider). The means of these segments were estimated for both the left and right sides. An increased CIMT was defined as >900 μm, and a carotid plaque was defined as a focal structure that intruded into the arterial lumen by ≥0.5 mm or 50% of the value of the neighbouring carotid IMT or that measured ≥1.5 mm from the media-adventitia boundary to the intima-lumen boundary [14].

The presence of LV hypertrophy was defined by electrocardiogram (ECG), echocardiography, or both methods. A standard 12-lead computerized resting ECG was obtained with the patient in the supine position. The Sokolow-Lyon index (SV1 + RV5/RV6, mm) was used to evaluate ECG-LV hypertrophy (>35 mm) [15]. Echocardiography was performed using the GE Vivid 7 ultrasound scanner (GE Vingmed ultrasound) with a 2.5-MHz linear transducer according to the American Society of Echocardiography recommendations [16]. The left ventricular mass (LVM) was calculated as follows: LV mass (g) = 0.8 {1.04[(LVEDD + PW + IVS)3 - (LVEDD)3]} + 0.6, in which LVEDD was the left ventricular end-diastolic diameter, and PW and IVS were the posterior wall thickness and the interventricular septum thickness at end diastole from an average of ≥3 cardiac cycles, respectively. Then, the LV mass was normalized for the body surface area (the DuBois formula: 0.007184 × height (cm)0.725 × weight (kg)0.425) [17] to obtain the LVM index (LVMI). LV hypertrophy echocardiography was defined as an LVMI >116 g/m2 for men and >95 g/m2 for women [18].

The LV ejection fraction was estimated based on a visual assessment of the left ventricular contractile performance and wall motion in multiple two-dimensional views. LV fractional shortening was calculated as (LVEDD - LVESD)/LVEDD, in which LVESD was the LV end-systolic diameter. LV systolic dysfunction was defined as LV fractional shortening <0.29 by M-mode, evidence of an ejection fraction <50% using the two-dimensional method, or both criteria [19].

The ankle-brachial index (ABI) was measured with an automatic waveform analyser (Colin Co., Komaki, Japan). Briefly, after taking at least a 5-min rest in the supine position, the subjects attached cuffs around their upper arms and ankles. The ABI was calculated as the ratio of the average SBP at the ankle of each leg divided by the average SBP in the arm. An ABI ≤0.9 was considered ABI-defined peripheral artery disease (PAD) [20].

### Statistical analysis

Continuous data were expressed as means ± standard deviations (SDs) and median with 25th and 75th percentiles. The data were compared using Student’s t-test for normally distributed variables and the Mann-Whitney U test for non-normally distributed variables. Categorical variables were expressed as percentages and were compared using the chi-square test.

We logarithmically transformed all subclinical biomarkers to normalize their skewed distributions. Subclinical atherosclerosis was defined as the presence of at least one component of the five subclinical disease markers: increased CIMT, presence of a carotid plaque, LV hypertrophy (by ECG or echocardiography), LV systolic dysfunction (by echocardiography), or reduced ABI. Then, we constructed a subclinical marker score ranging from 0 to 5 to calculate the number of the five abnormal measures. First, a linear regression model was used to evaluate the association between the CVH score (a continuous variable) and each subclinical biomarker and between the CVH score and each subclinical disease marker simultaneously stratified by gender. Standard regression beta (β) coefficients were calculated. We fitted 3 multivariate models. Model 1 had no adjustments. Model 2 adjusted for age and gender. Model 3 additionally adjusted for the education level. Next, we used a logistic regression model to assess the association between the numbers of subclinical disease marker components (subclinical marker score ≥ 1 vs. 0, ≥2 vs. 0 or 1, and ≥3 vs. 0, 1, or 2) and the CVH score (a continuous variable). The odds ratios (ORs) and 95% CIs were calculated. The adjusted models were the same as described above.

To examine the robustness of our results, sensitivity analyses were performed using a 14-point CVH score, which included 7 components based on the following categories: 0 = poor, 1 = intermediate, and 2 = ideal (Additional file 1). All statistical analyses were performed using the SPSS software version 20.0 (IBM). *P* values less than 0.05 were considered significant.

## Results

### General characteristics of the participants

A total of 3009 individuals were enrolled in this cross-sectional study. The mean age was 48.6 ± 9.7 years, and 33.5% of the participants were female. Table 1 shows the characteristics of the participants stratified by gender. The females tended to be older and had lower levels of education. The parameters, including the BMI, SBP, DBP, and FPG, and the proportions of participants who were current drinkers, had diabetes mellitus, and used diabetes medication were significantly higher in the males than in the females. Conversely, the high-density lipoprotein (HDL) levels were significantly higher in the females than in the males. The TC and low-density lipoprotein (LDL) values, the proportion of patients with hypertension, and the use of antihypertensive and lipid-lowering medications were not significantly different between the males and females.

**Table 1:** Clinical characteristics of the study sample

Characteristics	Overall (*n* = 3009)	Female (*n* = 1007)	Male (*n* = 2002)	*P* Value
Age, y	48.6 ± 9.7	49.9 ± 9.9	48.0 ± 9.6	<0.001
Education, n (%)				
Elementary school	206 (6.9)	135(13.4)	71 (3.6)	<0.001
High school	1353 (45.0)	538 (53.4)	815 (40.7)	
College or above	1450 (48.2)	334 (33.2)	1116 (55.7)	
Body mass index, kg/m^2^	24.9 ± 3.3	23.5 ± 3.1	25.5 ± 3.2	<0.001
Systolic blood pressure, mmHg	128.3 ± 16.6	125.5 ± 18.5	129.7 ± 15.3	<0.001
Diastolic blood pressure, mmHg	81.3 ± 11.3	76.3 ± 11.0	83.6 ± 10.8	<0.001
Hypertension, n (%)	409 (13.6)	125 (12.4)	284 (14.2)	0.195
Antihypertensive medication, n (%)	373 (12.4)	109 (10.8)	264 (13.2)	0.069
Fasting glucose, mmol/L	5.4 ± 1.4	5.2 ± 1.0	5.5 ± 1.6	<0.001
Diabetes mellitus, *n* (%)	134 (4.5)	22 (2.2)	112 (5.6)	<0.001
Diabetes medication, *n* (%)	111 (3.7)	21 (2.1)	90 (4.5)	<0.001
Total cholesterol, mmol/L	5.2 ± 1.0	5.2 ± 1.0	5.2 ± 1.0	0.199
HDL-cholesterol, mmol/L	1.4 ± 0.4	1.6 ± 0.4	1.3 ± 0.3	<0.001
LDL-cholesterol, mmol/L	2.9 ± 0.9	2.9 ± 0.9	2.9 ± 0.9	0.95
Lipid-lowering medication, *n* (%)	47 (1.6)	10 (1.0)	37 (1.8)	0.086
Cardiovascular health metrics				
Ideal diet, *n* (%)	102 (3.4)	51 (5.1)	51 (2.5)	0.001
Ideal smoking status, *n* (%)	1944 (64.6)	978 (97.1)	966 (48.3)	<0.001
Ideal physical activity, *n* (%)	339 (11.3)	112 (11.1)	227 (11.3)	0.859
Ideal BMI, *n* (%)	1591 (52.9)	726 (72.1)	865 (43.2)	<0.001
Ideal Blood pressure, *n* (%)	797 (26.5)	391 (38.8)	406 (20.3)	<0.001
Ideal Total cholesterol, *n* (%)	1472 (48.9)	517 (51.3)	955 (47.7)	0.060
Ideal Fasting glucose, *n* (%)	2258 (75.0)	826 (82.0)	1432 (71.5)	<0.001
Ideal CVH score	2.8 ± 1.3	3.6 ± 1.2	2.5 ± 1.2	<0.001
Subclinical biomarkers, median (Q1, Q3)				
Homocysteine, μmol/L	9.4 (7.6, 11.6)	7.8 (6.3, 9.3)	10.4(8.6, 12.5)	<0.001
C-reactive protein, mg/L	1.5 (0.8, 2.6)	1.3 (0.7, 2.4)	1.6 (0.8, 2.7)	<0.001
Microalbuminuria, mg/L	9.5 (5.3, 16.8)	7.0 (4.0, 13.0)	11.0 (6.0, 18.0)	0.005
Subclinical disease markers				
1. Increased CIMT, *n* (%)	422 (14.0)	118 (11.7)	30 (15.2)	0.010
2. Presence of carotid plaque, *n* (%)	805 (26.8)	250 (24.8)	555 (27.7)	0.097
3. LV hypertrophy by ECG/Echocardiography				
LV hypertrophy by ECG (Sokolow-Lyon criteria), *n* (%)	132 (4.4)	18(1.8)	114(5.7)	<0.001
LV mass-to-BSA ratio, g/m2	92.4 ± 18.2	77.8 ± 14.2	99.7 ± 15.3	<0.001
LV hypertrophy by Echocardiography, *n* (%)	341 (11.3)	84 (8.3)	257 (12.8)	<0.001
LV hypertrophy by ECG or Echocardiography, *n* (%)	453 (15.1)	100 (1.0)	353(17.6)	<0.001
4. LV systolic dysfunction by echocardiography				
Fractional shortening	0.37 ± 0.04	0.38 ± 0.04	0.37 ± 0.04	<0.001
LV systolic dysfunction, *n* (%)	63 (2.1)	11 (1.1)	52 (2.6)	<0.001
5. Peripheral arterial disease by ABI				
ABI ≤ 0.9, *n* (%)	35 (1.2)	19 (1.9)	16 (0.8)	0.011
Composite of subclinical disease markers				
At least 1, *n* (%)	1339 (44.5)	377 (37.4)	962 (48.1)	<0.001
At least 2, n (%)	383 (12.7)	105 (10.4)	278 (13.9)	
≥ 3, n (%)	56 (1.9)	16 (1.6)	40 (2.0)	
Mean score	0.59 ± 0.76	0.49 ± 0.72	0.64 ± 0.77	<0.001

*Q1*, quartile 1; *Q3*, quartile 3; *LDL*, low-density lipoprotein; *HDL*, high-density lipoprotein; *CVH*, cardiovascular health; *CIMT*, carotid intima-media thickness; *LV*, left ventricular; *BSA*, body surface area; *ECG*, electrocardiogram; *ABI*, ankle-brachial index

**Fig. 2:**
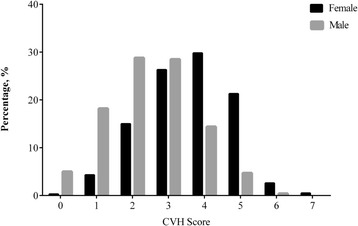
Distribution of cardiovascular health (CVH) scores in the females and males

The mean ± SD of the CVH score was 2.8 ± 1.3. Only 0.2% of the study participants had the 7 ideal CVH metrics (CVH score = 7), accounting for 0% of the males and 0.5% of the females. The CVH score distribution differed between the females and males. The males most frequently had CVH scores of 0 to 3, whereas the women most commonly had CVH scores of 4 to 7 (Fig. 2). The levels of the three subclinical biomarkers (homocysteine, CRP, and microalbuminuria) were all higher in the males than those in the females (all *P* < 0.01). This trend was consistent with the subclinical disease markers. The males had significantly greater percentages of increased CIMT, LV hypertrophy, and LV systolic dysfunction than the females. Conversely, carotid plaques did not differ by gender, and reduced ABI occurred less frequently in the males.

### Distribution of the subclinical biomarkers/subclinical disease markers by CVH category

The characteristics of the subclinical biomarkers/subclinical disease markers by poor, intermediate and ideal CVH statuses are reported in Table 2. When the CVH score increased from poor to ideal, the subclinical biomarker levels and the proportion of subclinical disease markers decreased significantly (all *P* < 0.05).

**Table 2:** Characteristics of subclinical impairments according to the CVH score categories

Characteristics	Poor CVH (0–2)	Intermediate CVH (3–4)	Ideal CVH (5–7)	*P* Value
Number, n	1238	1423	348	
Subclinical biomarkers, median (Q1, Q3)				
Homocysteine, μmol/L	10.2 (8.3,12.3)	9.0 (7.3, 11.0)	7.5 (6.2, 9.2)	<0.001
C-reactive protein, mg/L	1.9 (1.1, 3.2)	1.3 (0.7, 2.3)	0.9 (0.58, 1.6)	<0.001
Microalbuminuria, mg/L	11.6 (6.1, 21.6)	8.6 (4.9, 14.3)	5.2 (2.0, 9.8)	<0.001
Subclinical disease markers, *n* (%)				
1. Increased CIMT	214 (17.3)	184 (12.9)	24 (6.9)	<0.001
2. Presence of carotid plaque	396 (32.0)	364 (25.6)	45 (12.9)	<0.001
3. LV hypertrophy by ECG/echocardiography	215 (17.4)	211 (14.8)	27 (7.8)	<0.001
4. LV systolic dysfunction by echocardiography	39 (3.2)	23 (1.6)	1 (0.3)	0.001
5. Peripheral arterial disease by ABI ≤ 0.9	22 (1.8)	13 (0.9)	0	0.012
Composite of subclinical disease markers				
At least 1, *n* (%)	643 (51.9)	605 (42.5)	91 (26.3)	<0.001
At least 2, *n* (%)	201 (16.27)	167 (11.7)	15 (4.3)	<0.001
≥3, *n* (%)	31 (2.5)	24 (1.7)	1 (0.3)	0.021
Mean score	0.71 ± 0.80	0.56 ± 0.74	0.31 ± 0.56	<0.001

*Q1*, quartile 1; *Q3*, quartile 3; *CVH*, cardiovascular health; *CIMT*, carotid intima-media thickness; *LV*, left ventricular; *BSA*, body surface area; *ECG*, electrocardiogram; *ABI*, ankle-brachial index

### Association between the subclinical biomarkers and the CVH score

Table 3 shows the associations between each log-transformed subclinical biomarker level and the CVH score. We found that the homocysteine, CRP, and microalbuminuria concentrations were significantly inversely related to the CVH score (*P* < 0.001 for all). The associations remained robust after adjusting for gender, age, and education level. The stratified analyses found that this significant negative correlation was evident in both females and males (*P* < 0.001 for all).

**Table 3:** Associations between the CVH score (per 1-unit increase) and the log-transformed subclinical biomarker level stratified by gender

	Overall		Female		Male	
	β Coefficient (95% CI)	*P* Value	β Coefficient (95% CI)	*P* Value	β Coefficient (95% CI)	*P* Value
Homocysteine						
Model 1	−0.228 (−1.926 to −1.415)	<0.001	−0.188 (−1.827 to −0.935)	<0.001	−0.087 (−0.909 to −0.299)	<0.001
Model 2	−0.097 (−0.960 to −0.456)	<0.001	−0.115 (−1.271 to −0.412)	<0.001	−0.084 (−0.890 to −0.277)	<0.001
Model 3	−0.092 (−0.930 to −0.426)	<0.001	−0.111 (−1.242 to −0.388)	<0.001	−0.081 (−0.873 to −0.259)	<0.001
C-reactive protein						
Model 1	−0.233 (−0.815 to −0.603)	<0.001	−0.316 (−1.032 to −0.709)	<0.001	−0.177 (−0.621 to −0.378)	<0.001
Model 2	−0.190 (−0.674 to −0.479)	<0.001	−0.231 (−0.795 to −0.474)	<0.001	−0.175 (−0.617 to −0.373)	<0.001
Model 3	−0.182 (−0.652 to −0.457)	<0.001	−0.219 (−0.764 to −0.441)	<0.001	−0.172 (−0.605 to −0.363)	<0.001
Microalbuminuria						
Model 1	−0.296 (−0.929 to −0.729)	<0.001	−0.192 (−0.587 to −0.305)	<0.001	−0.244 (−0.677 to −0.476)	<0.001
Model 2	−0.228 (−0.734 to −0.546)	<0.001	−0.167 (−0.521 to −0.257)	<0.001	−0.245 (−0.676 to −0.475)	<0.001
Model 3	−0.224 (−0.720 to −0.534)	<0.001	−0.163 (−0.511 to −0.247)	<0.001	−0.238 (−0.662 to −0.463)	<0.001

Values are standard regression coefficient betas (95% CI) and p values. Each beta coefficient represents the change in log-biomarker per 1-unit increase in the CVH score. Model 1, unadjusted; model 2, adjusted for age and sex; model 3, adjusted for age, sex and level of education

### Association between the subclinical disease markers and the CVH score

An increased CIMT was present in 422 participants (14.0%), carotid plaque was present in 805 (26.8%), LV hypertrophy was present in 132 (15.1%), LV systolic dysfunction was present in 63 (2.1%), and peripheral arterial disease was present in 35 (1.2%). The mean ± SD of the subclinical disease marker score was 0.59 ± 0.76. Table 4 demonstrates a significant association between the CVH score (continuous variable) and each subclinical disease marker (*P* < 0.05 for all). Table 5 shows the association between the CVH score and the prevalence of the subclinical disease markers (categorical variable ≥1 vs. 0, ≥2 vs. 0 or 1, and ≥3 vs. 0, 1, or 2). We found that increasing CVH scores indicated a pattern of decreasing odds of the prevalence of subclinical disease markers (*P* < 0.001 for all). This negative correlation remained significant in the fully adjusted models and in the female and male subgroups, particularly in the females (*P* < 0.05 for all).

**Table 4:** Associations between the CVH score (per 1- unit increase) and each component of the subclinical disease markers stratified by gender

	Overall		Female		Male	
	β Coefficient (95% CI)	*P* Value	β Coefficient (95% CI)	*P* Value	β Coefficient (95% CI)	*P* Value
Increased CIMT						
Model 1	−0.115 (−0.580 to −0.305)	<0.001	−0.170 (−0.873 to −0.412)	<0.001	−0.076 (−0.412 to −0.110)	0.001
Model 2	−0.060 (−0.363 to −0.100)	<0.001	−0.087 (−0.551 to −0.104)	0.004	−0.071 (−0.407 to −0.085)	0.003
Model 3	−0.060 (−0.362 to −0.099)	<0.001	−0.081 (−0.527 to −0.082)	0.007	−0.072 (−0.527 to −0.082)	0.002
Presence of carotid plaque						
Model 1	−0.127 (−0.494 to −0.279)	<0.001	−0.261 (−0.902 to −0.566)	<0.001	−0.061 (−0.291 to −0.048)	0.006
Model 2	−0.071 (−0.324 to −0.108)	<0.001	−0.114 (−0.504 to −0.139)	0.001	−0.055 (−0.284 to −0.021)	0.023
Model 3	−0.069 (−0.315 to −0.100)	<0.001	−0.114 (−0.503 to −0.140)	0.001	−0.053 (−0.278 to −0.015)	0.029
LV hypertrophy by ECG/echocardiography						
Model 1	−0.118 (−0.648 to −0.348)	<0.001	−0.193 (−1.116 to −0.582)	<0.001	−0.062 (−0.391 to −0.067)	0.006
Model 2	−0.073 (−0.448 to −0.171)	<0.001	−0.141 (−0.872 to −0.366)	<0.001	−0.058 (−0.377 to −0.049)	0.011
Model 3	−0.066 (−0.419 to −0.141)	<0.001	−0.130 (−0.825 to −0.318)	<0.001	−0.053 (−0.363 to −0.033)	0.018
LV systolic dysfunction by echocardiography						
Model 1	−0.083 (−1.113 to −0.445)	<0.001	−0.105 (−1.945 to −0.506)	0.001	−0.059 (−0.802 to −0.119)	0.008
Model 2	−0.056 (−0.826 to −0.217)	0.001	−0.060 (−1.381 to −0.027)	0.042	−0.058 (−0.791 to −0.108)	0.010
Model 3	−0.053 (−0.803 to −0.195)	0.001	−0.059 (−1.358 to −0.012)	0.046	−0.056 (−0.778 to −0.096)	0.012
Peripheral arterial disease by ABI ≤ 0.9						
Model 1	−0.053 (−1.109 to −0.215)	0.004	−0.108 (−1.512 to −0.413)	0.001	−0.060 (−1.440 to −0.221)	0.008
Model 2	−0.065 (−1.218 to −0.406)	<0.001	−0.087 (−1.291 to −0.265)	0.003	−0.058 (−1.415 to −0.195)	0.010
Model 3	−0.062 (−1.182 to −0.317)	<0.001	−0.081 (−1.236 to −0.214)	0.005	−0.057 (−1.401 to −0.181)	0.011

Values are standard regression coefficient betas (95% CI) and *p* values. Each beta coefficient represents the change in log-biomarker per 1-unit increase in the CVH score. Model 1, unadjusted; model 2, adjusted for age and sex; model 3, adjusted for age, sex and level of education

**Table 5:** Associations between the CVH score (per 1- unit increase) and number of the subclinical disease markers stratified by gender

	Overall		Female		Male	
	OR (95% CI)	*P* Value	OR (95% CI)	*P* Value	OR (95% CI)	*P* Value
≥1 vs. 0						
Model 1	0.745 (0.704–0.788)	<0.001	0.533 (0.471–0.602)	<0.001	0.856 (0.797–0.902)	<0.001
Model 2	0.802 (0.749–0.858)	<0.001	0.676 (0.591–0.774)	<0.001	0.857 (0.791–0.928)	<0.001
Model 3	0.808 (0.755–0.865)	<0.001	0.682 (0.596–0.781)	<0.001	0.862 (0.796–0.933)	<0.001
≥2 vs. 0 or 1						
Model 1	0.703 (0.644–0.767)	<0.001	0.509 (0.423–0.613)	<0.001	0.742 (0.657–0.838)	<0.001
Model 2	0.724 (0.654–0.802)	<0.001	0.627 (0.513–0.766)	<0.001	0.744 (0.658–0.840)	<0.001
Model 3	0.729 (0.658–0.807)	<0.001	0.640 (0.523–0.782)	0.001	0.781 (0.700–0.872)	<0.001
≥3 vs. 0, 1, or 2						
Model 1	0.594 (0.463–0.761)	<0.001	0.465 (0.298–0.698)	<0.001	0.573 (0.422–0.778)	<0.001
Model 2	0.595 (0.464–0.763)	<0.001	0.568 (0.358–0.901)	0.016	0.579 (0.274–0.784)	<0.001
Model 3	0.603 (0.484–0.752)	<0.001	0.583 (0.368–0.925)	0.022	0.623 (0.468–0.830)	0.001

Values represent odds ratios (ORs) (95% confidence interval [CI]) per 1-unit increase in cardiovascular health (CVH) score; ≥ 1 denotes the presence of at least 1

component of increased carotid intima-media thickness, presence of carotid plaque, left ventricular hypertrophy (by ECG or echocardiography), left ventricular systolic dysfunction, or a reduced ankle-brachial index

Model 1, unadjusted; model 2, adjusted for age and sex; model 3, adjusted for age, sex and level of education

### Sensitivity analyses

An additional 14-point ideal CVH score was created. The analyses were repeated for both the subclinical biomarkers and the subclinical disease markers. The results were in accordance with the 7-point ideal CVH scores (Additional files 2, 3 and 4).

## Discussion

In this study, we investigated the relationships between subclinical biomarkers and subclinical disease markers of CVDs with ideal CVH metrics in a general urban population from central south China. We found that the CVH score was inversely related to both the biomarker levels (homocysteine, CRP, and microalbuminuria) and the presence of subclinical disease markers (increased CIMT, carotid plaques, LV hypertrophy, LV systolic dysfunction, and peripheral arterial disease). A similar negative correlation was observed in the female and male subgroups, and consistent trends were observed with the 14-point CVH score. To the best of our knowledge, this study is the first to correlate the relatively comprehensive subclinical profile of CVDs with ideal CVH in Chinese adults.

The biomarkers used in the study represent different biological pathways of atherosclerosis. CRP is the most clinically accepted inflammatory biomarker used to predict future cardiovascular events in asymptomatic individuals [21]. Hyperhomocysteinaemia has multiple potentially toxic effects on atherogenesis, including damaging endothelial cells, reducing the flexibility of vessels, and adversely affecting the process of homeostasis [22, 23]. Microalbuminuria is predictive of worsening microvascular disease in the kidney, reflects a generalized abnormality of vascular function, and is associated with a strong risk of increasing CVDs [24]. Notably, the items in the CVH metrics are risk factors associated with the biomarkers. BMI, smoking, and diabetes are associated with CRP [25]. Smoking, lack of exercise, and hypertension are related to hyperhomocysteinaemia [26]. Diabetes, hypertension, smoking, blood lipids, BMI, and dietary protein are correlated with microalbuminuria [27]. The observed associations of the selected biomarkers with the CVH scores suggest that a range of biological pathways can mediate the favourable effects of the CVH score on subclinical atherosclerosis or disease.

The subclinical stage serves as an intermediate endpoint for the risk evaluation and optimal treatment. We used previously validated markers of subclinical disease. An increased CIMT, carotid plaques and reduced ABI were indicators for macrovascular disease (coronary heart disease, myocardial infarction, PAD and stroke), whereas LV hypertrophy and LV systolic dysfunction were indicators of target organ damage. Among the composite of the subclinical disease markers, the majority of the participants (55.5%) had no subclinical disease markers, only 12.7% had at least 2 components, and 1.9% had ≥3 components, which was consistent with a previous study [9]. There may be several reasons for this status. First, the median age in our cohort was relatively young (48.6 years) with regard to CVD risk. Second, the prevalence of hypertension and diabetes was low, implying a lower CVD risk. Third, many CVH metrics (e.g., mean lipid, fasting glucose, and BP values) were inside the normal ranges, suggesting that the subjects were either healthy or well treated.

Our study found that subclinical impairments were independently associated with low CVH scores. Low CVH scores may primarily contribute to chronic subclinical damage, leading to CVDs via subclinical impairments. Previous studies have largely focused on the association of subclinical stages with each individual behaviour or health factor [28]. However, many of these factors coexist or occur in aggregation and thus may be interwoven into multiple biologically related pathways. The association in the present study reflected the combined and cumulative effects of exposure to risk factors on subclinical lesions and thus provided more accurate information compared to exploring each factor individually.

Previous studies also uncovered associations between CVH and subclinical impairments. In the Paris Prospective Study III, more ideal CVH metrics were positively associated with a lower presence of subclinical disease markers of carotid structure and function (common CIMT, carotid plaques, lower Young’s elastic modulus, and carotid dispensability coefficient) among community subjects aged 50 to 75 years [10]. In the Young Finns Study, Laitinen et al. reported that a greater number of average lifetime CVH metrics and favourable changes in the CVH status in childhood or young adulthood were associated with a reduced risk of coronary artery calcification, carotid dispensability, and increased CIMT in middle age [8]. In the Framingham study, higher ideal CVH scores were inversely related to a composite measure of subclinical CVDs (including higher carotid artery IMT or stenosis, LV systolic dysfunction, LV hypertrophy, microalbuminuria, or reduced ABI) [9]. The Special Turku Coronary Risk Factor Intervention Project for Children [STRIP] study reported that healthy CVH scores were directly associated with a lower aortic intima-media thickness and greater elasticity in adolescents [29]. Another study indicated that improvements in ideal cardiovascular health through midlife to late life were associated with a lower CVD prevalence and better cardiac structure and vascular functions, including carotid-femoral and brachial-ankle PWV (baPWV), in the elderly [30]. Similar results were obtained by Yan et al. in a Chinese population after assessing the association between the CVH score and baPWV [31]. Our study findings have confirmed and extended these results in Asian populations.

A global ideal CVH was rare (0.2%) in our study, which was consistent with other studies with more representative samples (values of approximately 0.1 to 0.2% were reported in American and Chinese urban populations) [32–34]. Females had better CVH profiles and a lower prevalence of most subclinical disease markers than males, and the relationship between the CVH score and markers tended to be stronger among females than that among males, which was also in line with prior reports [35]. The exact mechanism underlying this observed gender difference warrants further investigation. Generally, females may be less exposed to cardiovascular risk factors, such as smoking, alcohol use and overweight, and they may receive more protective effects from endogenous oestrogens prior to reaching the premenopausal age [36, 37]. Considering the above findings, we should attach great concerns to the primary care of CVDs, especially for males. In our study, women were more likely to be classified with a reduced ABI, which was consistent with previous epidemiological evidence showing that women had lower ABI values than men [38, 39]. Gender differences in the ABI value may occur because men on average are taller than women, and men have a greater tendency to experience medial arterial calcinosis, which may avoid an ABI decline [40]. Although the gender differences in PAD must be explored, the intrinsic differences in the ABI criterion and measurements may confound the results [41].

The major strengths of this study include the large population-based sample, the use of a wide range of biomarkers and markers of subclinical data, and the performance of sensitivity analyses. However, some limitations to the present study should also be acknowledged. First, the cross-sectional design of our study only allowed the documentation of one-time sampling data that did not take the time-associated changes in the CVH scores into consideration, which might have improved the accuracy of our findings. Similarly, we did not explore the association between the CVH score and CVD incidence through prospective analyses. Second, some potential biomarkers, such as aldosterone, B-type natriuretic peptide (BNP), and growth differentiation factor-15 (GDF-15), and markers such as coronary artery calcium and lipoprotein-associated phospholipase A2 (Lp–PLA2) were not involved in these observed associations. Third, socioeconomic factors, such as family incomes, paying for medical expenses, frequency of health care visits, and the duration of treatment (antihypertensive, lipid-lowering diabetes and medication), might contribute to the relationship between CVH metrics and subclinical impairments, but we did not have this information. Finally, we used modified definitions of physical activity and diet that varied from the exact AHA definition to compute the scores for the CVH metrics.

## Conclusion

In summary, the prevalence of ideal CVH was low in the adult population in central south China. The CVH score was associated with a favourable profile of subclinical biomarkers or markers of CVDs, thereby supporting the use of the CVH score as a reasonable and useful tool for assessing and preventing subclinical impairments, thus reducing the burden of CVDs. These findings may deepen our understanding of the biological mechanisms and processes underlying CVH and CVDs.

## Additional files

Additional file 1: Table S1. Creation of the 14-point CVH score. (DOCX 52 kb)

Additional file 2: Table S2. Associations between the 14-point CVH score (per 1-unit increase) and the log-transformed subclinical biomarker level stratified by gender. (DOCX 48 kb)

Additional file 3: Table S3. Associations between the 14-point CVH score (per 1-unit increase) and each component of the subclinical disease markers stratified by gender. (DOCX 51 kb)

Additional file 4: Table S4. Associations between the 14-point CVH score (per 1- unit increase) and number of the subclinical disease markers stratified by gender. (DOCX 50 kb)

## Abbreviations

ABI: ankle-brachial index
BMI: body mass index
CIMT: carotid intima-media thickness
CRP: C-reactive protein
CVDs: cardiovascular diseases
CVH: ideal cardiovascular health
DBP: diastolic blood pressure
FPG: fasting plasma glucose
HDL: high-density lipoprotein
IVS: interventricular septum thickness
LDL: low-density lipoprotein
Lp–PLA2: lipoprotein-associated phospholipase A2
LV: left ventricular
LVEDD: left ventricular end-diastolic diameter
LVESD: left ventricular end-systolic diameter
LVM: left ventricular mass
PAD: peripheral arterial disease
PW: posterior wall thickness
SBP: systolic blood pressure
SD: standard deviation
TC: total cholesterol
