# Supplementary material for: Ideal cardiovascular health and the subclinical impairments of cardiovascular diseases: a cross-sectional study in central south China
Source: BMC Cardiovasc Disord. 2017 Oct 18;17:269. doi: 10.1186/s12872-017-0697-9 (PMC5648483; doi:10.1186/s12872-017-0697-9)
Supplement: Supplementary file 1 — Creation of the 14-point CVH score. (DOCX 52 kb) [file 12872_2017_697_MOESM1_ESM.docx]

**Additional file 1: Table S1**. **Creation of the 14-point CVH scorea**

| Metrics | Poor | Intermediate | Ideal |
| --- | --- | --- | --- |
| Current smoking status | current | former or quit <12 months ago | never or quit >12 months ago |
| Body mass index | ≥30 kg/m2 | 25-29.9 kg/m2 | <25 kg/m2 |
| Physical activity | sedentary or light activity | 1–149 min/week moderate intensity or 1–74 min/week vigorous intensity | ≥150 min/week moderate intensity or ≥75 min/week vigorous intensity |
| Healthy diet scoreb | 0–1 components | 2-3 components | 4-5 components |
| Serum total cholesterol | ≥6.2 mmol/L[240 mg/dl] | 5.2-6.1 mmol/L[201-239 mg/dl] or treated to goal | untreated<5.2mmol/L[200 mg/dL] |
| Blood pressure | ≥140/90 mmHg | 120–139/80–89 mmHg or treated to goal | untreated <120/80 mmHg |
| Fasting plasma glucose | ≥11.1mmol/L[126 mg/dl] | 5.6-11.0 mmol/L[100–125 mg/dl] or treated to goal | untreated< 5.6mmol/L[100 mg/dL] |

aA previously defined scoring system were assigned to each category of the seven metrics and summed: ideal=2 points, intermediate=1 point,and poor=0 point, for a total score ranging from 0 to 14 points[1].

bHealthy diet score: 1. with cereals and legumes as the basic food; 2. ≥ 500 g (1 “Jin”) vegetables and fruits/day; 3. < 100 g (2 “Liang”) red meat/day; 4. regular (in most weeks) intake of unprocessed fish and/or soybean products; 5. preference for non-salty food.

[1] Unger E, Diez-Roux AV, Lloyd-Jones DM, Mujahid MS, Nettleton JA, Bertoni A, Badon SE, Ning H, Allen NB. Association of neighborhood characteristics with cardiovascular health in the multi-ethnic study of atherosclerosis[J]. Circulation. Cardiovascular quality and outcomes, 2014, 7(4): 524-531.
