# Supplementary material for: Ideal cardiovascular health and the subclinical impairments of cardiovascular diseases: a cross-sectional study in central south China
Source: BMC Cardiovasc Disord. 2017 Oct 18;17:269. doi: 10.1186/s12872-017-0697-9 (PMC5648483; doi:10.1186/s12872-017-0697-9)
Supplement: Supplementary file 2 — Associations between the 14-point CVH score (per 1-unit increase) and the log-transformed subclinical biomarker level stratified by gender. (DOCX 48 kb) [file 12872_2017_697_MOESM2_ESM.docx]

**Additional file 2 : Table S2. Associations between the 14-point CVH score (per 1-unit increase) and the log-transformed subclinical biomarker level stratified by gender**

|  | Overall | | | Female | | Male | |
| --- | --- | --- | --- | --- | --- | --- | --- |
|  | β Coefficient (95% CI) | *P* Value | | β Coefficient (95% CI) | *P* Value | β Coefficient (95% CI) | *P* Value |
| Homocysteine | | | | | | | |
| Model 1 | -0.222(-3.143 to -2.291) | | ＜0.001 | -0.164(-2.599 to -1.190) | ＜0.001 | -0.093(-1.642 to -0.592) | ＜0.001 |
| Model 2 | -0.100(-1.640 to -0.793) | | ＜0.001 | -0.106(-1.914 to -0.524) | 0.001 | -0.092(-1.617 to -0.573) | ＜0.001 |
| Model 3 | -0.090 (-1.528 to-0.691) | | ＜0.001 | -0.099 (-1.830 to -0.463) | 0.001 | -0.085 (-1.537 to -0.495) | ＜0.001 |
| C-reactive protein | | | | | | | |
| Model 1 | -0.249(-1.434 to -1.084) | | ＜0.001 | -0.312(-1.606 to -1.097) | ＜0.001 | -0.209(-1.209 to -0.794) | ＜0.001 |
| Model 2 | -0.214(-1.246 to -0.919) | | ＜0.001 | -0.248(-1.334 to -0.817) | ＜0.001 | -0.207(-1.218 to -0.802) | ＜0.001 |
| Model 3 | -0.202 (-1.185 to -0.860) | | ＜0.001 | -0.228 (-1.243 to -0.728) | ＜0.001 | -0.203 (-1.184 to -0.772) | ＜0.001 |
| Microalbuminuria | | | | | | | |
| Model 1 | -0.328(-1.696 to -1.366) | | ＜0.001 | -0.219(-1.025 to -0.582) | ＜0.001 | -0.282(-1.311 to -0.971) | ＜0.001 |
| Model 2 | -0.264(-1.391 to -1.079) | | ＜0.001 | -0.200(-0.945 to -0.520) | ＜0.001 | -0.282(-1.312 to -0.971) | ＜0.001 |
| Model 3 | -0.256 (-1.353 to -1.043) | | ＜0.001 | -0.188 (-0.899 to -0.480) | ＜0.001 | -0.275 (-1.283 to-0.945) | ＜0.001 |

Values are standard regression coefficient betas (95% CI) and p values. Each beta coefficient represents the change in log-biomarker per 1-unit increase in the CVH score. Model 1, unadjusted; model 2, adjusted for age and sex; model 3, adjusted for age, sex and level of education.
