# Supplementary material for: Ideal cardiovascular health and the subclinical impairments of cardiovascular diseases: a cross-sectional study in central south China
Source: BMC Cardiovasc Disord. 2017 Oct 18;17:269. doi: 10.1186/s12872-017-0697-9 (PMC5648483; doi:10.1186/s12872-017-0697-9)
Supplement: Supplementary file 3 — Associations between the 14-point CVH score (per 1-unit increase) and each component of the subclinical disease markers stratified by gender. (DOCX 51 kb) [file 12872_2017_697_MOESM3_ESM.docx]

Additional file 3: Table S3. Associations between the 14-point CVH score (per 1-unit increase) and each component of the subclinical disease markers stratified by gender

|  | Overall | | Female | | | | Male | | |
| --- | --- | --- | --- | --- | --- | --- | --- | --- | --- |
|  | β Coefficient (95% CI) | *P* Value | β Coefficient (95% CI) | | *P* Value | | β Coefficient (95% CI) | *P* Value | |
| Increased CIMT | | | | | | | | | |
| Model 1 | -0.098 (-0.858 to -0.400) | ＜0.001 | | -0.153 (-1.275 to -0.548) | ＜0.001 | | -0.072 (-0.699 to -0.154) | | 0.008 |
| Model 2 | -0.060 (-0.608 to -0.165) | 0.001 | | -0.087 (-0.880 to -0.158) | 0.005 | | -0.070 (-0.687 to -0.136) | | 0.003 |
| Model 3 | -0.060 (-0.602 to -0.165) | 0.001 | | -0.077 (-0.811 to -0.101) | 0.012 | | -0.059 (-0.607 to -0.089) | | 0.002 |
| Presence of carotid plaque | | | | | | | | | |
| Model 1 | -0.120 (-0.787 to -0.428) | ＜0.001 | | -0.247 (-1.357 to -0.827) | ＜0.001 | -0.076 (-0.586 to -0.135) | | | 0.002 |
| Model 2 | -0.090 (-0.608 to -0.165) | ＜0.001 | | -0.139 (-0.910 to -0.322) | 0.001 | -0.069 (-0.550 to -0.103) | | | 0.004 |
| Model 3 | -0.085 (-0.602 to -0.165) | ＜0.001 | | -0.139 (-0.904 to -0.327) | 0.001 | -0.061 (-0.499 to -0.084) | | | 0.006 |
| LV hypertrophy by ECG/echocardiography | | | | | | | | | |
| Model 1 | -0.110 (-1.026 to -0.525) | ＜0.001 | | -0.183 (-1.687 to -0.846) | ＜0.001 | | -0.059 (-0.657 to -0.096) | | 0.009 |
| Model 2 | -0.074 (-0.753 to -0.286) | ＜0.001 | | -0.142 (-1.389 to -0.570) | ＜0.001 | | -0.057 (-0.639 to -0.083) | | 0.011 |
| Model 3 | -0.058 (-0.644 to -0.181) | ＜0.001 | | -0.122 (-1.250 to -0.439) | ＜0.001 | | -0.045 (-0.568 to -0.008) | | 0.044 |
| LV systolic dysfunction by echocardiography | | | | | | | | | |
| Model 1 | -0.085 (-1.885 to -0.772) | ＜0.001 | | -0.104 (-3.041 to -0.777) | 0.001 | | -0.063 (-1.420 to -0.252) | | 0.005 |
| Model 2 | -0.061 (-1.465 to -0.442) | ＜0.001 | | -0.068 (-2.348 to -0.161) | 0.025 | | -0.063 (-1.427 to -0.257) | | 0.005 |
| Model 3 | -0.056 (-1.382 to -0.372) | 0.001 | | -0.065 (-2.278 to -0.129) | 0.028 | | -0.058 (-1.352 to -0.193) | | 0.009 |
| Peripheral arterial disease by ABI ≤ 0.9 | | | | | | | | | |
| Model 1 | -0.076 (-2.269 to -0.905) | ＜0.001 | | -0.122 (-2.583 to -0.857) | ＜0.001 | -0.069 (-2.681 to -0.591) | | | 0.002 |
| Model 2 | -0.070 (-2.139 to -0.792) | ＜0.001 | | -0.106 (-2.316 to -0.661) | ＜0.001 | -0.068 (-2.644 to -0.578) | | | 0.002 |
| Model 3 | -0.061 (-2.021 to -0.533) | 0.001 | | -0.096 (-2.158 to -0.529) | ＜0.001 | -0.066 (-2.598 to -0.528) | | | 0.003 |

Values are standard regression coefficient betas (95% CI) and p values. Each beta coefficient represents the change in log-biomarker per 1-unit increase in the CVH score. Model 1, unadjusted; model 2, adjusted for age and sex; model 3, adjusted for age, sex and level of education.
