# Supplementary material for: Ideal cardiovascular health and the subclinical impairments of cardiovascular diseases: a cross-sectional study in central south China
Source: BMC Cardiovasc Disord. 2017 Oct 18;17:269. doi: 10.1186/s12872-017-0697-9 (PMC5648483; doi:10.1186/s12872-017-0697-9)
Supplement: Supplementary file 4 — Associations between the 14-point CVH score (per 1- unit increase) and number of the subclinical disease markers stratified by gender. (DOCX 50 kb) [file 12872_2017_697_MOESM4_ESM.docx]

Additional file 4: Table S4. Associations between the 14-point CVH score (per 1- unit increase) and number of the subclinical disease markers stratified by gender

|  | Overall | | Female | | Male | |
| --- | --- | --- | --- | --- | --- | --- |
|  | OR (95% CI) | *P* Value | OR (95% CI) | *P* Value | OR (95% CI) | *P* Value |
| ≥1 vs. 0 | | | | | | |
| Model 1 | 0.847 (0.819 – 0.876) | ＜0.001 | 0.692 (0.641– 0.747) | ＜0.001 | 0.859 (0.854 – 0.937) | ＜0.001 |
| Model 2 | 0.863 (0.829 – 0.898) | ＜0.001 | 0.777 (0.715 – 0.844) | ＜0.001 | 0.901 (0.860 – 0.945) | ＜0.001 |
| Model 3 | 0.870 (0.835 – 0.906) | ＜0.001 | 0.782 (0.719 – 0.851) | ＜0.001 | 0.914 (0.876 – 0.953) | ＜0.001 |
| ≥2 vs. 0 or 1 | | | | | | |
| Model 1 | 0.822 (0.775 – 0.872) | ＜0.001 | 0.671 (0.602 – 0.749) | ＜0.001 | 0.843 (0.786 – 0.903) | ＜0.001 |
| Model 2 | 0.827 (0.786 – 0.869) | ＜0.001 | 0.737 (0.656 – 0.829) | ＜0.001 | 0.850 (0.793 – 0.912) | ＜0.001 |
| Model 3 | 0.832 (0.784 – 0.883) | ＜0.001 | 0.754 (0.670 – 0.849) | 0.001 | 0.882 (0.829 – 0.938) | ＜0.001 |
| ≥3 vs. 0, 1, or 2 | | | | | | |
| Model 1 | 0.789 (0.686 – 0.906) | ＜0.001 | 0.698 (0.554 – 0.879) | 0.002 | 0.781 (0.659 – 0.924) | 0.004 |
| Model 2 | 0.798 (0.694 – 0.917) | ＜0.001 | 0.703 (0.555 – 0891) | 0.004 | 0.787 (0.664 – 0.933) | 0.006 |
| Model 3 | 0.810 (0.718 – 0.912) | ＜0.001 | 0.746 (0.585 – 0.953) | 0.019 | 0.833 (0.714 – 0.971) | 0.019 |

Values represent odds ratios (ORs) (95% confidence interval [CI]) per 1-unit increase in cardiovascular health (CVH) score; ≥1 denotes the presence of at least 1 component of increased carotid intima-media thickness, presence of carotid plaque, left ventricular hypertrophy (by ECG or echocardiography), left ventricular systolic dysfunction, or a reduced ankle-brachial index.

Model 1, unadjusted; model 2, adjusted for age and sex; model 3, adjusted for age, sex and level of education.
